# Colorectal Lesion diagnosis using transformer and deep learning with multiscale feature interface

**DOI:** 10.1371/journal.pone.0357664

**Published:** 2026-09-03

**Authors:** Dhirendra Prasad Yadav, Bhisham Sharma, Julian L. Webber, Abolfazl Mehbodniya

**Affiliations:** 1 Department of Computer Engineering & Applications, G.L.A. University, Mathura, Uttar Pradesh, India; 2 Centre for Research Impact & Outcome, Chitkara University Institute of Engineering and Technology, Chitkara University, Rajpura, Punjab, India; 3 Department of Electronics and Communication Engineering, Kuwait College of Science and Technology (KCST), Kuwait City, Kuwait; Chunghwa Telecom Co. Ltd., TAIWAN

## Abstract

Colorectal cancer is the third most common malignancy worldwide. Manual screening requires expertise and resources. However, advancements in AI (artificial intelligence) have reduced the computation burden and time. Machine and deep learning have recently been used to diagnose colorectal lesions. The requirement of handcrafted features makes machine learning models expertise-dependent. At the same time, classical CNN (convolutional neural network) miss the global attention of the features. This work presents CDCTNet (colorectal diagnosis convolution transformer network), a hierarchical model for colorectal disease detection. Our model utilized two convolution blocks for the local high-dimensional spatial features from the lesion. In addition, the ViT encoder is used in parallel with the CNN block to provide a global correlation of the feature map. Furthermore, we designed an IEM block for the interaction of the features between the convolution block and ViT encoder to improve the attention on the features. The CDCTNet is evaluated on Kather and Kvasir datasets and obtained a precision and Kappa score of 96.60% and 95.02%, respectively. At the same time, CDCTNet has recall and F1 scores of 98.08% and 97.94%.

## 1. Introduction

The latest guidelines for colorectal cancer screening emphasize the need for an early start in the management of death statistics since it is highly curable if diagnosed early. Colorectal cancer, the third most common malignancy, affects more than 1.9 million patients and causes nearly 900,000 deaths worldwide annually [1]. Established methods of screening, including colonoscopy, stool-based tests, and advanced imaging, have shown to have significantly reduced mortality. The recent WHO global cancer data brings before the public eye that there is disparity in regions over the utilization of cancer services, as screening services are still quite limited to low-income countries. These disparities will inevitably lead to diagnoses diagnosed at later stages and more deaths mainly due to the underserved populations [2].

Colorectal cancers are abnormal growths appearing on parts of the large intestine: the colon and rectum. These tumors can either be benign or malignant. Polyps represent examples of benign types, whereas malignant cases present as colorectal cancer [3]. The majority of cases originate as a polyp which, left untreated for long periods, then turns malignant. For a successful treatment, early detection is essential. Molecular testing, advanced imaging studies, and non-invasive screening tests are just some of the techniques used to detect colorectal cancer. The screening techniques include two types of FOBT: FIT is a more sensitive version that does not require any dietary restrictions, whereas gFOBT does require dietary restrictions. The Stool DNA Test (Cologuard) tests stool for mutations in DNA that would indicate advanced polyps or cancer. Since sigmoidoscopy only visualizes the lower portion of the colon, endoscopic studies such as colonoscopy allow direct visualization of the entire colon. EUS is useful in the evaluation of rectal cancer and essential for understanding the scope of tumor invasion [4].

Imaging greatly helps the staging and especially diagnosis of colorectal cancer. Although MRI is utilized for precise staging of rectal cancers, the virtual colonoscopy or CT colonography permits non-invasive visualization of the colon and rectum. Older techniques include X-ray visualization with the Lower GI series or Barium Enema, while PET scans are employed through metabolic activity to detect metastasis and recurrence [5]. By molecular and genetic testing, the tumors genetic makeup can be established; further tests are Mismatch Repair Deficiency (dMMR) and Microsatellite Instability (MSI). Recently, a new technology known as liquid biopsy has the capability to literally trail cancer without intervention directly into the system. It tracks through DNA or tumor cells present in the bloodstream. These methods make possible for accurate diagnosis, early detection, and proper treatment of colorectal cancers [6].

Machine learning (ML) and deep learning (DL) are finding key roles in the detection and classification of CRC. Such advanced techniques can analyze large datasets like medical images, histopathological slides, genetic data, and clinical records to identify those patterns that predict cancerous growths [7,8]. In histopathology, ML models can analyze digitized biopsy slides classified into tissue forms like benign, pre-cancerous, or malignant, and pathologists can diagnose more accurately. Colonoscopy employs DL models, especially CNNs, to classify video in real-time for the identification of polyps or early-stage tumors, hence improving dramatically detection rates by identifying lesions otherwise likely to be missed in the process of visual review [9]. Finally, deep learning techniques like CNNs and U-Nets are applied in the segmentation of tumors in medical imaging, for instance, CT or MRI, hence enabling the accurate localization and staging of the tumor. Similarly, these have been applied in genomic data for the prediction of patient outcomes and treatment responses in association with genetic mutations like KRAS or MSI status. Since deep models are often data hungry, transfer learning could be well-applied to enable even the smallest domain-specific datasets at their disposal and hence enhancing the accuracy of detection. New applications of ML and DL models include predicting patient survival and recurrence risk, guiding a course of treatment, and even a personalized treatment strategy [10]. The hurdles of data availability and model interpretability will allow AI technologies to be successfully incorporated into clinical workflows, so opportunities for more precise and efficient CRC detection, diagnosis, and treatment should contribute better to patient outcomes and facilitate the development of personalized medicine [11].

The requirement of handcrafted features makes machine learning model expertise dependent. At the same time, classical CNN (convolutional neural network) miss the global attention of the features. This work presents CDCTNet (colorectal diagnosis convolution transformer network), a hierarchical model for colorectal disease detection. Our model utilized two convolution blocks for the local high-dimensional spatial features from the lesion. In addition, the ViT encoder is used in parallel with the CNN block to provide a global correlation of the feature map. Furthermore, we designed an IEM block for the interaction of the features between the convolution block and ViT encoder to improve the attention on the features. The CDCTNet is evaluated on Kather and Kvasir datasets.

The contribution of the propose method is as follows.

(1) We combine CNN and ViT to leverage the colorectal lesion’s local spatial features and global contextual information.
(2) We strengthen the feature interaction between CNN and ViT using IEM block. To achieve this, spatial feature extracted from the CNN block is projected for the embedding space using dense projection. After that, contribution of the CNN and ViT feature is optimized using learnable fusion parameter before performing the multi-head attention in the ViT encoder.
(3) To evaluate model performance, we utilized the Kather and Kvasir dataset and achieved precision and Kappa score of 96.60% and 95.02%, respectively.

Remainder of the manuscript is organized as follows.

The colorectal diagnosis is summarized in section 2. At the same time, section 3 explains the architecture of CDCTNet. Moreover, section 4 provides quantitative results, and section 5 provides a detailed discussion. Finally, in section 6, we present the conclusion of the proposed method with future scope and limitations.

## 2. Literature Review

Using MSI and dMMR in colorectal tumor cells on routine histology slides, this study developed a faster and cheaper deep-learning system compared to molecular assays. From the slides, the model trained a deep-learning detector that could classify samples as MSI; cross-validation was used to evaluate the model’s performance for N = 6406 specimens, and an external cohort of n = 771 specimens validated the results. It identified dMMR-positive tissues in a large global validation cohort at an AUROC of 0.96. This technology has potential to be translated into low-cost, high-throughput assessment of colorectal tissue samples [12].

This identification of mutations within the KRAS gene is considered a key factor in devising specific treatments for patients diagnosed with CRC. Therefore, this paper applied pre-treatment CE-CT imaging to test the model’s predictive performance by estimating the status of KRAS mutation in CRC patients using a DL process with a ResNet. model recruited a total of 157 CRC patients whose pathology was confirmed. These patients were divided into two parts, namely: training with a total of n = 117 and a testing with n = 40 [13]. CRC screening commonly used tool is colonoscopy. They train a deep learning model called CRCNet on 464,105 images from 12,179 patients to use in the optical diagnosis of colorectal cancer. Then we test the model on three distinct datasets of 2263 patients. AUPRC of CRCNet patients is 0. While better than average endoscopists, during achieving comparable recall rate on one test set and precision on the other two, CRCNet has performed with 93.7% versus 83.8%; p = 0.02 for one test set precision and recall rate across two test sets were 91.3% versus 83.8%; two-sided t-test, p < 0.001 and 96.5% versus 90.3%; p = 0.006 [14].

Based on histology, deep learning can accurately predict the status of lymph nodes in colorectal cancer. Routine histological WSIs from the primary tumors of 2431 patients of the DACHS may be utilized for prediction of LNM of CRC based on the deep-learning model. The external test set consisted of WSIs and data from 582 patients. The SBAIP has an AUROC of 71.0% on the internal test set. Clinical classification gave an AUROC of 67.0%. The grouping of the two classifiers gives an AUROC of 74.1%. The SBAIP’s performance fell to the level of 61.2% on the TCGA set but the clinical classifier’s presentation remained stable. The impact of the T stage was quite strong on the clinical classifier [15]. The article provides a DL network that describes the morphological change of the tumor in order to assess the response of patients. The model tested 1,028 patients enrolled prospectively in the VELOUR trial (NCT00561470). In mCRC, model found the DL network outperforming its size-based counterpart in forecasting the early on-treatment response: CIndex: 0.649, p = 0.009, z-test. Compared with size/DL based-only models (all p < 0.001, z-test), the prediction performance to C-Index: 0.694 could be further improved by integrating DL network with size-based methodology [16].

Using whole-slide images of stained hematoxylin and eosin colorectal cancer slides, the study aimed to establish a novel pipeline of deep learning as a potential alternative for the prevalent tests that predicted the grade of significant molecular pathways and mutations. It used 502 slides consisting of the primary colorectal tumours from 499 patients in the colon and rectal cancer. Model 1, ResNet18, classified the tumour from the non-tumour. These tumour slides were then summed up to the ResNet34-tuned model 2. The iterative draw and rank sampling method used to generate mean AUROCs. For CIMP-high status, the mean AUROC was 0·79 (SD 0·05) [17].

The researchers designed and validated a novel deep learning model using AI that can help in screening colorectal specimens for possible cancers. As such, this would improve the sensitivity of cancer detection and classification. The study cohort comprised the WSI of 294 colorectal specimens. To achieve instance segmentation, Model combined a ResNet-101 feature extractor with a deep learning model based on the Faster Region Based Convolutional Neural Network architecture. The hybrid AI-model was trained with a larger set of 105 resection WSIs. Our results were validated in a set of 150 biopsies WSIs by comparing them to the classifications of two blinded pathologists working on their own. With a sensitivity of 97.4% to detect high-risk dysplasia and cancer characteristics, the AUC for the validation cohort of the AI model was 0.917 [18].

Based on diagnostic codes of PHC consultations, an SGB-based model has been constructed to predict the presence or absence of NMCRC. For the SGB model, 361 variables were used as predictors, out of which 184 had nonzero influence. Out of them, 16 variables together hold a combined normalised relative influence of 63.3% with an NRI of >1%. The combined NRI of variables on bleeding and anaemia stood at 27.6%. The specificity of the model stood at 83.5%, while its sensitivity at 73.3%. The largest odds ratios of marginal effects were associated with changes in bowel habits, at 28.8 [19]. Candidate lipid biomarkers in the plasma samples from each of the CRC patients were analyzed for biomarker levels through HR-LC-MS that was subsequently integrated with chemokine, gene, and clinical information. For classifying samples from stage, I to III CRC and CLM patients and control subjects who are either cancer-free or patients with polyps/diverticulitis, Bayesian neural net and multilinear regression-machine learning identified candidate biomarkers. This suggests that the precise lipid signature and chemokines, such as platelet factor-4 and interluken-8; IL-8 could be used to enhance prognostic accuracy [20].

That involved a massive data set used, and the study was dietary-based CRC study with 109,343 participants. The nine supervised and unsupervised machine learning algorithms were assessed using the combined dataset. Good performance of supervised and unsupervised models was demonstrated in predicting phenotypes of CRC and non-CRC. An ANN-based model emerged as the best algorithm, with a 1% rate of misclassification of CRC and a 3% misclassification rate of non-CRC [21]. Recent works demonstrated the potential of CNNs for retrieval of relevant biomarkers from whole slide images obtained by microscope. Biomarkers based on CNNs had predictive patient outcomes for colorectal cancer similar to the above-mentioned gold standards. In this work, model refined an existing CNN model using novel training strategy and the obtained model outperforms all previously known methods. Figure 4 contains the results of the model on the external test set, average accuracy equals to 95.6%, and on the internal test set, equaling to 99.5%. The proposed method is the first of its kind that applies interpretability techniques and had reduced errors, particularly in biomarker-relevant classes, like lymphocytes [22].

Authors propose the use of ML-based models, particularly DNN, for a faster calibration by increasing the emphasis on the use of ML. The CRC-AIM model simulates the following types of events: Adenoma generation, Growth, Transition to cancer, CRC survival, based on the natural history of colorectal cancer. The trained DNN gave good predictive accuracy with average mean squared error of 0.014 and 0.016 for training data and testing data, respectively [23]. The authors introduce a weakly supervised deep learning framework by merging the classic S-DL features with SNA features extracted from cell networks. The study uses the TCGA-CRC-DX dataset, which comprises 502 diagnostic slides from primary colorectal tumors and 499 patients. The average AUPRC and AUROC of the proposed method lie in the range from 2.4% to 4% and 7% to 8.8% respectively while predicting mutations in the TP53 gene, BRAF gene, HM, and CIN [24].

The authors also intend to design a CAD system, called Color-CADx, that combines the use of three CNN architectures: ResNet50, DenseNet201, and AlexNet, which can improve the classification accuracy. The paper uses two freely available datasets for training and validating: NCT-CRC-HE-100K Dataset and Kather_texture_2016_image_tiles. The best accuracy obtained was 99.3% with the first one, which turns out to be a remarkable performance for the classification of CRC. The correct rate of the model for the second one was 96.8% [25]. Articles seek to explore how clinical and demographic information can be utilized using multiple machine learning (ML) models for the prediction of metastasis in colorectal cancer patients. Among 1,127 CRC patients treated at Taleghani Hospital, 183 had metastases and are thus part of the dataset. The top performers in this exercise were the NN and RF algorithms, which when applied to the balanced dataset indicated an area under the curve (AUC) of 100%, sensitivity of 100%, and accuracy well above 99%. Thus, the best models for metastases prediction in CRC patients are NN and RF [26]. The summary of the performance and dataset is presented in Table 1.

**Table 1:** Summary of the methods and their performance on different datasets.

Authors	Model	Types of Images	Dataset	Performance
Echle et al. [12]	Deep learning	Microsatellite instability status, histological images	Private dataset: 7177 images	Precision: 95%
He et al. [13]	ResNet	CT imaging, KRAS mutation status	Private dataset: 157 images	Sensitivity: 89%
Zhou et al. [14]	CRCNet	Optical diagnostic images, colorectal cancer status	Private dataset: 464,105 images	Precision: 93.7%
Kiehl et al. [15]	Deep CNN	Histology images, lymph node status	Private: DACHS Public: TCGA	AUROC: 74.10%
Lu et al. [16]	GoogleNet+LSTM	Serial medical imaging, early treatment response	Private dataset: 1028 patients	C-Index = 0.649
Bilal et al. [17]	ResNet18 + ResNet34	Histology images, molecular pathway status	Public: TCGA-CRC-DX	AUROC = 0.79
Ho et al. [18]	ResNet101 + Faster R-CNN	Histopathological images, colorectal cancer status	Private: 294 WSIs (plus 105 resection WSIs for training and 150 biopsy WSIs for validation	AUC = 0.917
Nemlander et al. [19]	Stochastic Gradient Boosting (SGB)	Primary care data, metastatic status	Private: Clinical records	Specificity = 83.5%
Krishnan et al. [20]	Bayesian Neural Network	Lipid biomarkers, staging status	Private: Plasma samples from CRC patients	Accuracy:78%
Rahman et al. [21]	ANN	Dietary data, colorectal cancer status	Public: 109,343 participants	misclassification of CRC of 1%
Prezja et al. [22]	CNN	Histological tissue decomposition	Private: 100,000 images	Accuracy:95.6%
Vahdat et al. [23]	Deep Learning model	Microsimulation model inputs	Public: CRC-AIM Microsimulation Dataset	MSE = 0.014 (training)
Zamanitajeddin et al. [24]	Color-CADx	Cell network analysis, molecular pathway status	Public: TCGA-CRC-DX	AUROC = 99%
Sharkas et al. [25]	ResNet50 + DenseNet201 + AlexNet	Histological images, colorectal cancer status	Public: NCT-CRC-HE-100K & Kather_Texture_2016	Accuracy = 96.8%
Talebi et al. [26]	Neural Network (NN) & Random Forest (RF)	Clinical data, metastasis status	Private (Hospital Clinical Dataset) 1,127 patients	AUC = 100%
Fu et al. [27]	ViT	Whole slide images (WSIs)	Private: 875 images	Accuracy: 94.80%
Li et al. [28]	DMFI-Net	Endoscopic images	Public: Kvasir	Accuracy: 87.67%

## 3. Proposed method

**Fig 1:**
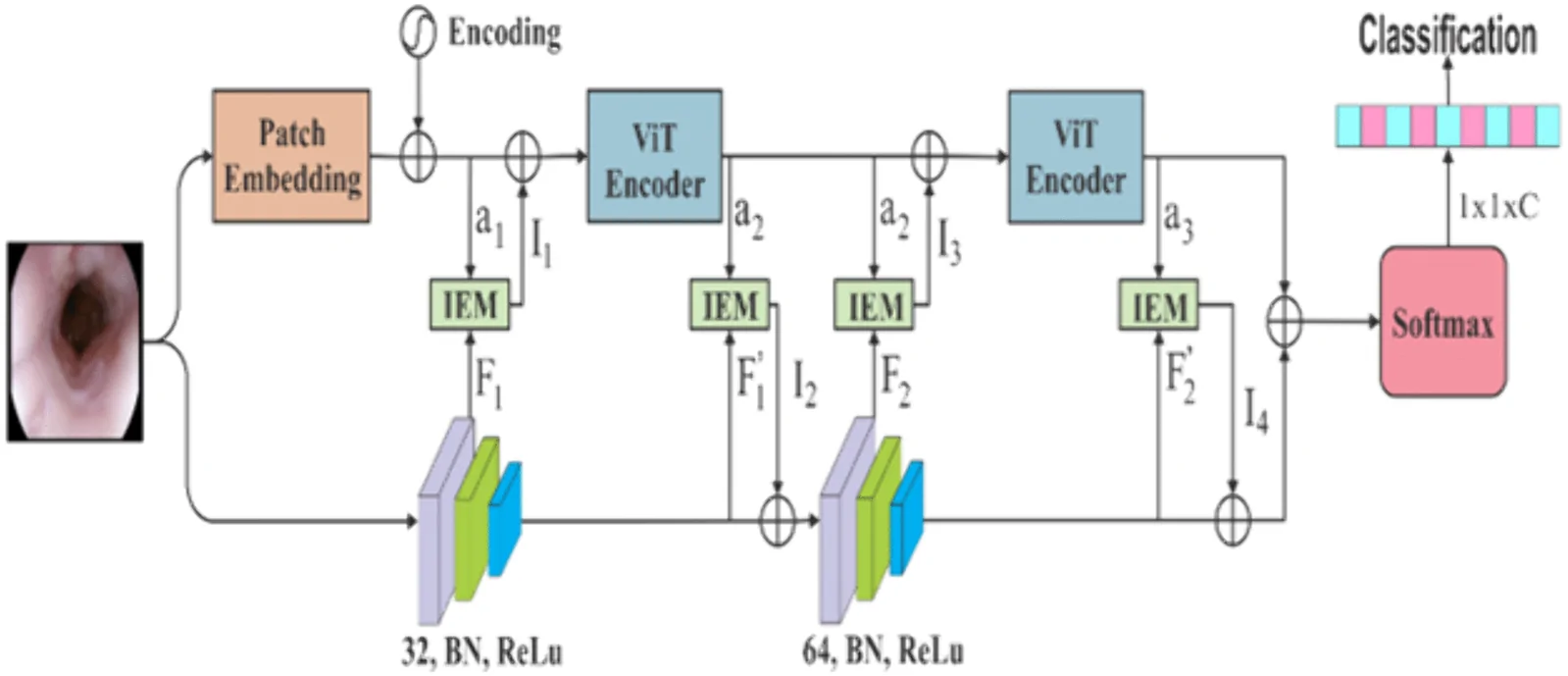
The architecture of CDCTNet having convolution block, IEM and ViT enoder for colorectal cancer diagnosis.

The architecture of the CDCTNet (colorectal diagnosis convolution transformer network) is shown in Fig 1. The CDCTNet consists of two convolution blocks that extract local spatial features and two transformer encoder blocks that provide global attention to contextual information. Furthermore, information interaction between the transformer and convolution block is controlled by the IEM (information exchange module). The feature extracted from both blocks is fused and passed through a global average pooling (GAP) layer. Finally, classification is performed by a softmax layer.

Let the input image $x\in R^{H\times W\times C}$, where H, W, and C are height, width and channel be fed to the convolution block (Conv1) consisting of convolution size 32 followed by the max-pooling of 2x2 for the local spatial feature. The extracted feature map is passed to the second convolution block (Conv2), which has a convolution size 64 and a max-pooling layer of size 2x2. The overall activation of j^th^ feature map at spatial position (u, v) of the i^th^ layer is aggregated as $Z_{i,j}$ and calculated as follows.


$$\begin{array}{c} Z_{i,j}^{u,v}|=\mu (b_{i,j}+\sum_{k=\text{1}}^{F_{k-\text{1}}\sum }\sum_{m=-\alpha }^{\alpha \sum }\sum_{n=-\beta }^{\beta \sum }w_{i,j,k}^{n,m_{i-\text{1},k}^{u+\beta ,v+\alpha }} \end{array}$$ (1)

Where, $b_{i,j}$=Bias term, $w_{i,j}$  = j^th^ feature map of the i^th^ layer, μ= ReLU activation, 2α+1 = Height of the Kernel and 2β+1=Width of the kernel.

### 3.1. The ViT

The ViT is known for its capability long range feature co-relation in the image. We divided the input image into patches of size 16x16, after that positional embedding is performed by projecting to dimension d as follows.


$$\begin{array}{c} Y=PE(linear(x^{\prime }) \end{array}$$ (2)

Where PE(.)= Positional embedding and linear(.)= Linear embedding in dimension d. After that, attention for each head is calculated using Q (query), K (key) and V (value) as follows.


$$\begin{array}{c} A=Softmax\left(\frac{QK^{T}}{\sqrt{d}}\right)V \end{array}$$ (3)

Where $Q=YW_{Q},K=YW_{K},V=YW_{V}$ and d = Embedding dimension. Furthermore, the self-attention (A) for all head (h) is normalized and concatenated to calculate MHSA (multi-head self-attention) as follows.


$$\begin{array}{c} M=Concat(A_{\text{1}},A_{\text{2}},....A_{h})W_{\text{0}}. \end{array}$$ (4)

$$\begin{array}{c} M^{\prime }=LN(M), \end{array}$$

$$\begin{array}{c} M^{{\;}^{\prime \prime }}=FFN(M^{\prime })+M^{\prime } \end{array}$$ (5)

### 3.2. The IEM block

**Fig 2:**
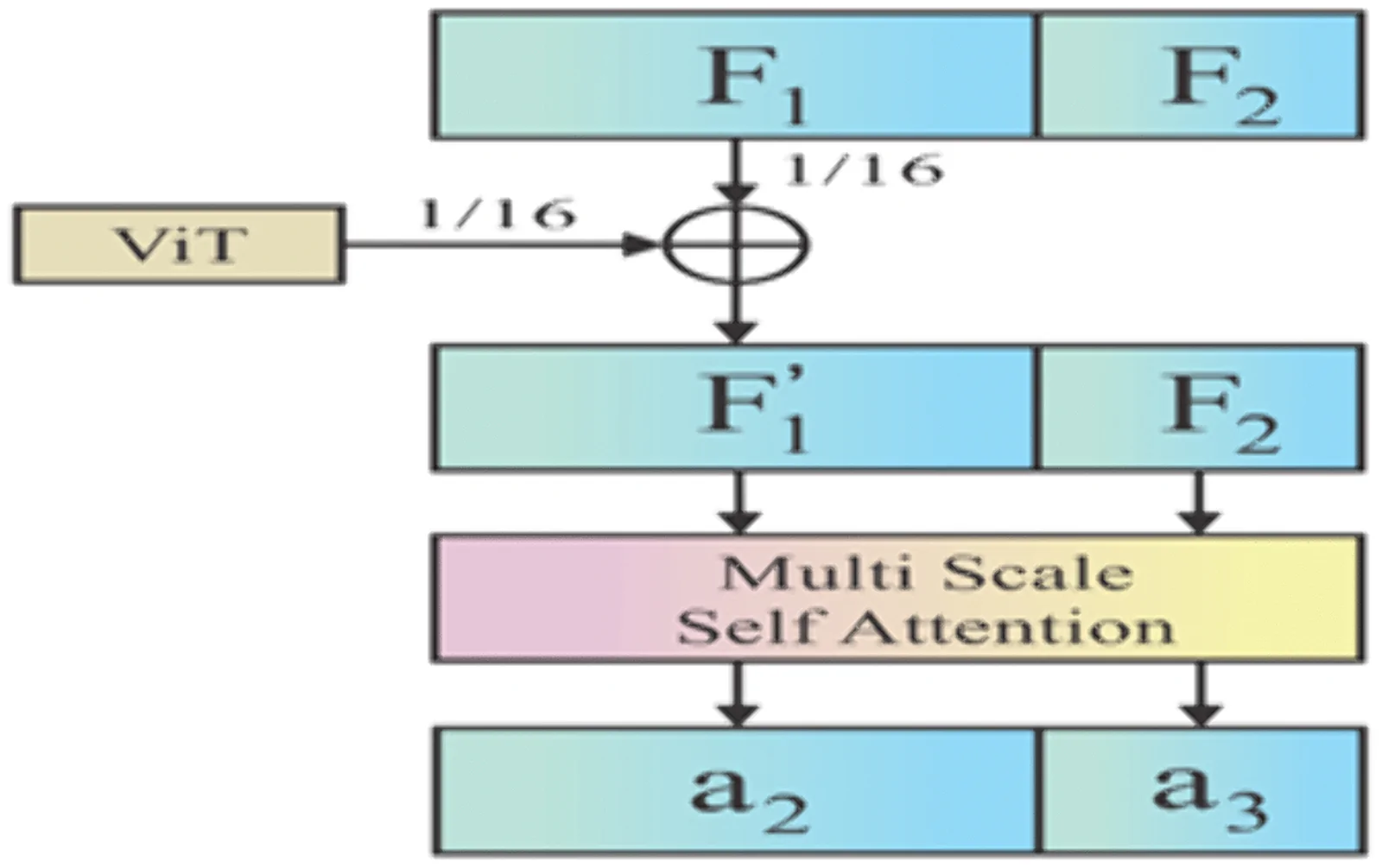
Illustration of the IEM block used for bidirectional information exchange.


The IEM block provide path for the interaction of the high dimensional spatial features obtained from the CNN blocks and global attention map of ViT block shown in Fig 2. Let the feature map obtained from the Cov1 is $F_{\text{1}}\in R^{H_{\text{1}}\times W_{\text{1}}\times C_{\text{1}}}$ and $F_{\text{2}}\in R^{H_{\text{2}}\times W_{\text{2}}\times C_{\text{2}}}$ from Conv2. Furthermore, we assume output of the ViT encoder block is $T_{out}\in R^{N\times D}$. We flattened the feature maps of the ConV1 and Conv2 block as follows.

$$\begin{array}{c} F_{\text{1}}^{\prime }=Flatten\left(F_{\text{1}}\right), \end{array}$$

$$\begin{array}{c} F_{\text{2}}^{\prime }=Flatten(F_{\text{2}}) \end{array}$$ (6)

Further, the dimension of the convolution and ViT encoder block is different. We applied dense layer to align the dimensions as follows.


$$\begin{array}{c} F_{\text{1}}^{{\;}^{\prime \prime }}=Dense(F_{\text{1}}), \end{array}$$

$$\begin{array}{c} F_{\text{2}}^{{\;}^{\prime \prime }}=Dense(F_{\text{2}}), \end{array}$$

$$\begin{array}{c} T_{out}^{\prime }=Dense(T_{out}) \end{array}$$ (7)

Here, $F_{\text{1}}^{{\;}^{\prime \prime }},F_{\text{2}}^{{\;}^{\prime \prime }},T_{out}\in R^{N\times D}$, N = Number of tokens, and D = Embedding dimension. In the experiment we set N = 361 and D = 384 for the kather dataset. In addition, the value of N and D are 1444 and 384, respectively for the KvasirV2 dataset. After this we fused the convolutions and. ViTs encoder block features as follows.


$$\begin{array}{c} F_{fused\text{1}}=\beta T_{out}^{\prime }+F_{\text{1}}^{{\;}^{\prime \prime }}, \end{array}$$

$$\begin{array}{c} F_{fused\text{2}}=\lambda T_{out}^{\prime }+F_{\text{2}}^{{\;}^{\prime \prime }}, \end{array}$$ (8)

Here α,β=Learnable scalar which balance the contribution of the ViT encoder during fusion of spatial feature and we set its value range between 0 and 1. The fused feature obtained from each scale is passed to the MHSA block to provide attention to the spatial features as follows.


$$\begin{array}{c} A_{\text{1}}=Softmax\left(\frac{F_{fused\text{1}}W_{Q}(F_{fused\text{1}}W_{K}{)}^{T}}{\sqrt{d}}\right)(F_{fused\text{1}}W_{V}), \end{array}$$

$$\begin{array}{c} A_{\text{2}}=Softmax\left(\frac{F_{fused\text{2}}W_{Q}(F_{fused\text{2}}W_{K}{)}^{T}}{\sqrt{d}}\right)(F_{fused\text{2}}W_{V}) \end{array}$$ (9)

Finally, we aggregated the features obtained from the convolution and ViT encoder blocks using concatenation as follows.


$$\begin{array}{c} F_{agg}=Concat(A_{\text{1}},A_{\text{2}}) \end{array}$$ (10)

After that, a GAP (global average pooling) layer is utilized in the model to get the fixed size feature map for the classification as follows.


$$\begin{array}{c} F=\frac{\text{1}}{N}\sum_{j=\text{1}}^{N}F_{agg}[j] \end{array}$$ (11)

Here $F\in R^{\text{2}D}$= Final feature vector after aggregation of the feature map, N = Number of tokens. Finally, a softmax layer is used for disease classification as follows.


$$\begin{array}{c} C_{i}=\frac{e^{F_{i}}}{\sum_{j=\text{1}}^{K}e^{F_{j}}} \end{array}$$ (12)

Where $F_{i}$=logits vector obtained from GAP layer, K = Number of classes. We set K = 8 for the Kaither and KvasirV2 datasets.

### 3.3 The hybrid loss function

We combine the strength of categorical_crossentropy and isotropic loss function to regularize the model's training. The categorical_crossentropy penalized the divergence between the predicted probabilities and GT (ground truth) labels for correctly predicting colorectal disease. However, it does not constrain the learned feature space geometry, which can lead to poor generalization for diverse visual feature-based disease images. To overcome the challenge, we integrated isotropic loss, which computes cosine similarity terms to reduce rotation invariance between predicted and reference feature maps. In addition, it captures translation invariance through Euclidian distance term to reduce absolute displacement in the feature map of the Kaither and KaiserV2 datasets. We calculated the categorical_crossentropy loss as follows.


$$\begin{array}{c} L_{C}=-\frac{\text{1}}{N}\sum_{j=\text{1}}^{K}y_{j}log(p_{j}) \end{array}$$ (13)

Where y = True label, p = Predicted probability and K = Number of classes. Furthermore, we computed the cosine similarity and Euclidian distance between the true feature vector and predicated as follows to develop isotropic loss function.


$$\begin{array}{c} L_{I}=\beta \cdot \left(\text{1}-\frac{F\cdot F_{\text{1}}}{\left\|F\right\|\left\|F_{\text{1}}\right\|}\right)+\alpha \left\|F-F_{\text{1}}\right\| \end{array}$$ (14)

Where, F= True feature vector, the true feature vector denotes the reference of the ground truth classes., $F_{\text{1}}$=Predicted feature vector, predicted feature is embedding feature map obtained by the softmax layer, after global average pooling β,α = Hyperparmeter. Finally, the hybrid loss function is defined as follows.


$$\begin{array}{c} L_{CI}=L_{C}+\eta L_{I} \end{array}$$ (15)

Where η = Learnable parameter. The algorithm for colorectal disease diagnosis using CDCTNet is as follows.

**Algorithm:** Colorectal Lesion diagnosis Using CDCTNet.

**Input:** Image $x\in R^{H\times W\times C}$

**Output:** Predicted label $y^{\prime }\in R^{C}$, where C = Number of classes in the dataset (Kather or KvasirV2)

(1) Resize image and set BS = 32, Epochs = 140, Initial learning rate = 0.0001, Patch size = 8x8

(2) for i = 1–140 do,

 (a) Extract spatial features using Eq. (1)

 (b) Calculate Q, K, and V as follows

  $Q=YW_{Q}$

   $K=YW_{K}$

   $V=YW_{V}$

 (c) Find MHSA using Eq. (4)

 (d) Pass the information between CNN and ViT using IEM

 (e) Train the CDCTNet

end

(3) Calculate performance measures

(5) Plot the training and validation loss

## 4. Results

The results on the Kather and Kvasir datasets of the CDCTNet and SOTA methods are described below.

### 4.1. Dataset

We evaluated model performance on two open-source datasets. The KvasirV2 dataset is collected using endoscopic equipment in Norwey through Vestre Viken Health Trust (VV) at Baerum hospital. All the images in the dataset are annotated by experts from the Cancer Registry of Norway (CRN). Furthermore, the KvasirV2 dataset, contains images of varying resolutions ranging from 720x576 to 1920x1072 pixels stored in JPEG file format. Furthermore, it contains 8 classes, including dyed-lifted-polyps (DLP), dyed-resection-margins (DRM), esophagitis (ESO), normal-cecum (NOC), normal-pylorus (NOP), normal-z-line (NZL), polyps (POL) and ulcerative-colitis (ULC). Each class contains an equal number of 1000 images [29]. The second dataset, Kather, stained with hematoxylin and eosin (H&E) provided by the Heidelberg University, Mannheim, Germany contains 5000 histopathological slide images divided into 8 classes. Each class contains 625 images with a resolution of 150x150 pixels and is stored in TIF file format. The eight classes include TUMOR, STROMA, COMPLEX, LYMPHO, DEBRIS, MUCOSA, ADIPOSE and EMPTY [30]. The KvasirV2 dataset contains macroscopic colonoscopic images, whereas, Kather contains microscopic histopathology patches. The texture and structure patterns of the datasets’ modalities impose a significant challenge for the model to precisely diagnose the disease.

### 4.2. Experimental settings

We conducted each experiment on NVIDIA QUADRO RTX-4000 GPU having 128GB RAM. Python 3.11 is used to write and execute the scripts on the Windows 10 operating system. Furthermore, the Adam optimizer accelerates the training with an initial learning rate 0.0001. Moreover, the CDCTNet is trained for 140 epochs in a batch size 32. In the KvasirV2 dataset, images contain macroscopic colonoscopic images that may produce frame similarity and potential data leakage. To overcome the problem, we utilized patient-level dataset splitting and selected 80% per class for the training and 20% for the validation. At the same time, the Kather dataset includes histopathological images obtained by cropping the independent whole slide of different patients. Therefore, in the Kather dataset evaluation, we applied class-stratified random sampling and divided the 80% and 20% for training and validation.

### 4.3. Quantitate results

**Fig 3:**
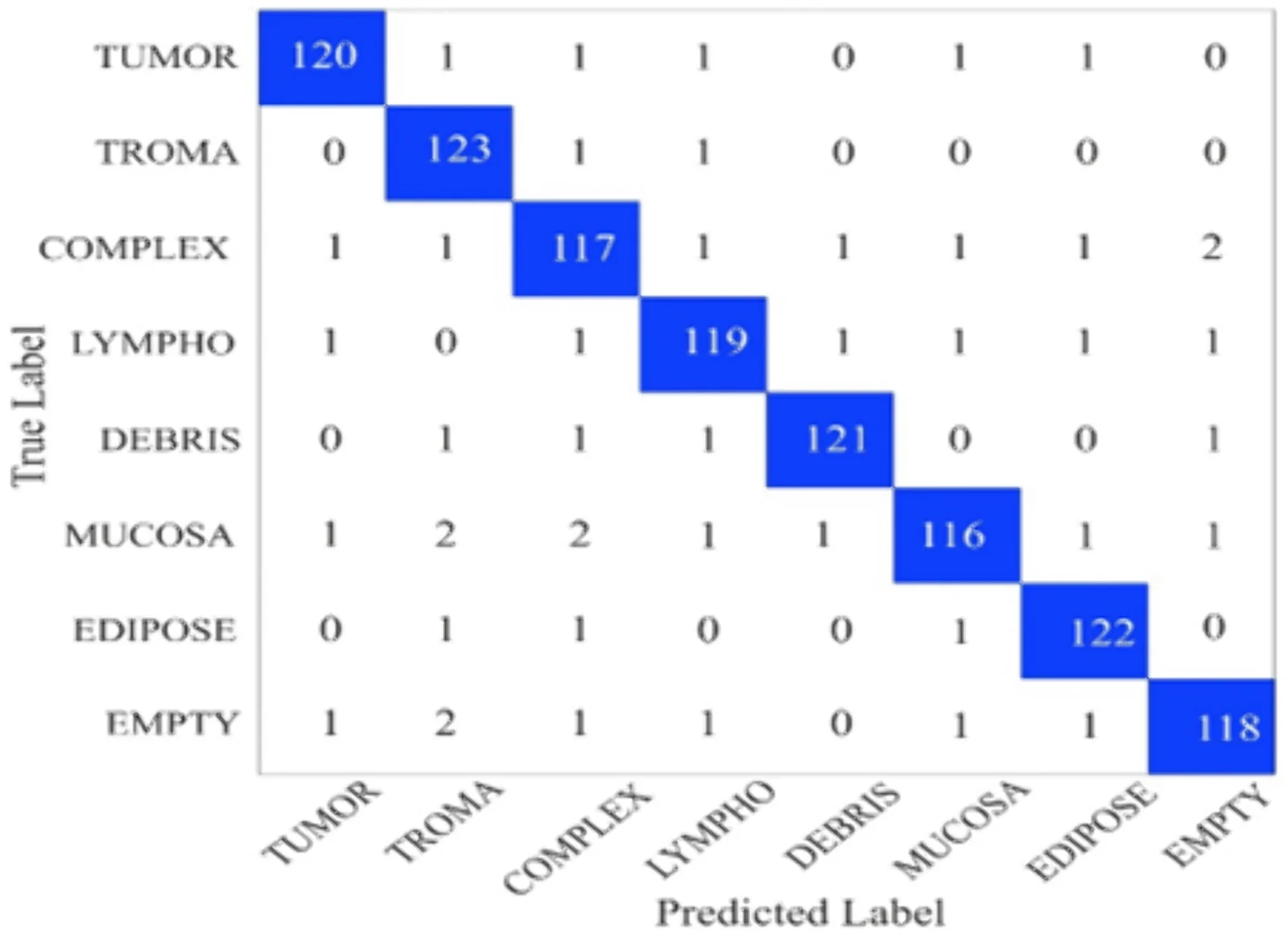
Confusion matrix of the Kather dataset on the validation set.

**Fig 4:**
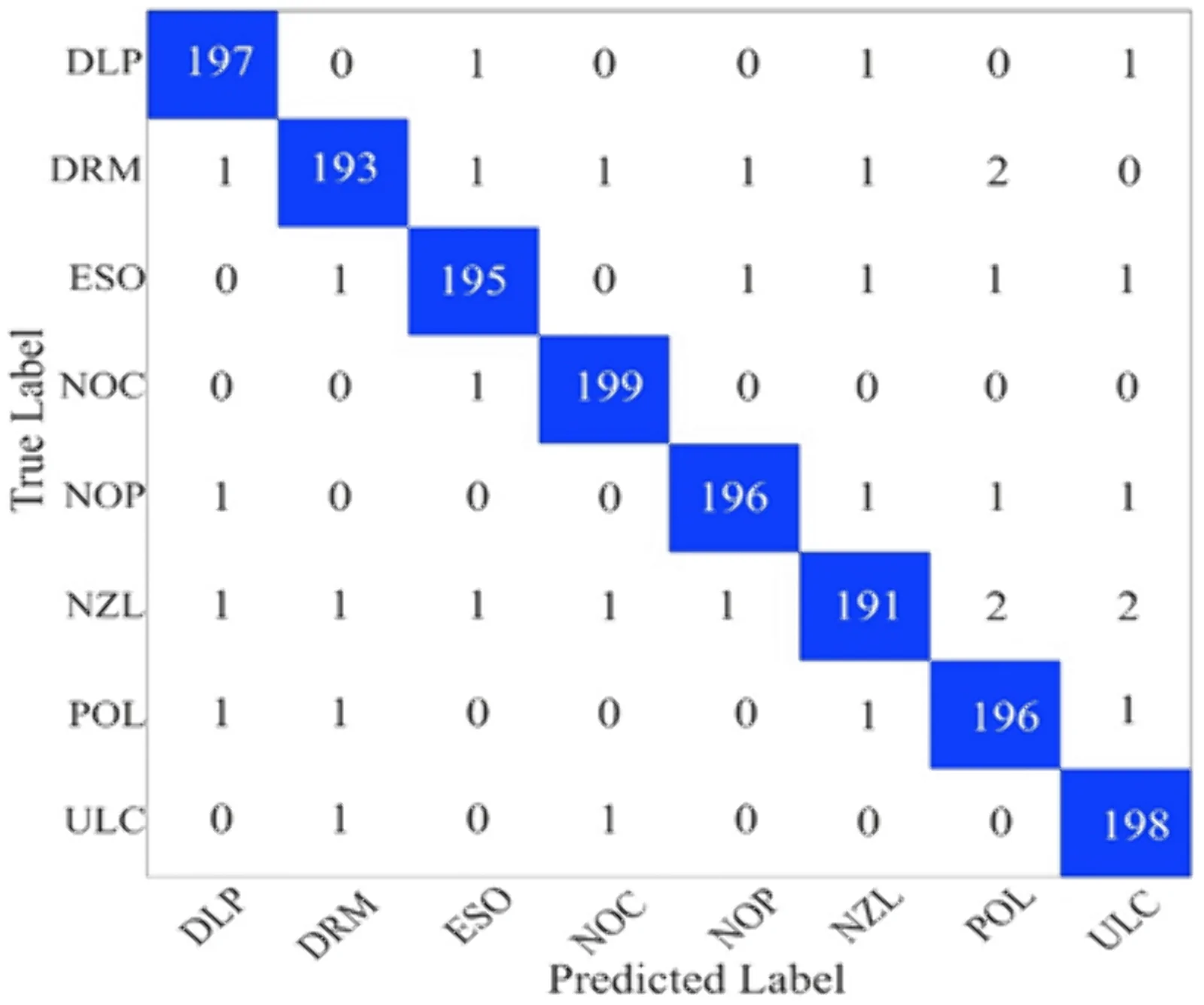
Confusion matrix of the KvasirV2 dataset on the validations set.

This section presents the experimental results on the KvasirV2 and Kather datasets. For the model’s training and validation, datasets are randomly divided into 80% and 20%, respectively. Images of the KvasirV2 dataset are resized to 300x300 pixels, and the Kather dataset image resolution is the same as in the dataset. After the training model is validated, the confusion matrix of the Kather and KvasirV2 datasets is shown in Fig 3 and Fig 4, respectively. Fig 3 shows the model has 20 false positive and 24 false negative values. Meanwhile, on the KvasirV2 dataset CDCTNet has 21 false positive and 14 false negative values.

After plotting confusion matrix performance measures precision, recall, F1-score, accuracy and kappa is calculated shown in Table 2. Table 2 shows that model achieved precision and Kappa value of 96.60% and 95.02% respectively. At the same time model obtained F1-score and recall value of 97.94% and 98.08% respectively on the KvasirV2 dataset.

**Table 2:** Quantitative results on the Kather and KvasirV2 datasets.

Dataset	Precision	Recall	F1-score	Accuracy	Kappa
Kather	96.60	95.61	96.10	95.60	95.02
KvasirV2	97.81	98.08	97.94	97.81	97.50

## 5. Discussion

In this section we present the comparative study with state-of the-art (SOTA) methods and ablation study conducted on different components of the model.

### 5.1. Comparative study with SOTA methods

We compare the proposed model performance on Kather and KvasirV2 with ResNet50 [31], Inception V3 [32], MobileNetV3 [33], YOLOV9 [34], MnasNet [35], and CellViT [36]. Furthermore, for a fair comparison, the experimental settings were kept the same, and the result is depicted in Table 3 and Table 4. Table 3 shows that MobileNetV3 and Inceptionv3 precision values are 78.70% and 84.43%, respectively. The recall values of the ResNet50 and YOLOV9 are very close. At the same time, MnasNet and CellViT F1-score are 91.79% and 93.99%, respectively. Moreover, our method achieved the highest F1-score and recall values of 96.10% and 95.60%, respectively.

**Table 3:** Comparison with SOTA on the Kather dataset.

Method	Precision	Recall	F1-score	Accuracy	Kappa
ResNet50	87.62	88.92	88.27	88.82	89.42
InceptionV3	84.43	86.14	85.28	83.85	84.50
MobileNetV3	78.70	80.25	79.47	80.18	79.67
YOLOV9	90.29	89.79	90.04	91.34	90.98
MnasNet	92.12	91.46	91.79	93.16	92.78
CellViT	94.26	93.72	93.99	94.02	94.27
Proposed	96.60	95.61	96.10	95.60	95.02

**Table 4:** Comparison with SOTA methods on the Kvasirv2 dataset.

Method	Precision	Recall	F1-score	Accuracy	Kappa
ResNet50	90.25	92.49	91.36	90.21	91.82
InceptionV3	87.70	89.16	88.42	88.24	89.13
MobileNetV3	84.30	85.19	84.74	86.04	85.18
YOLOV9	92.92	91.84	92.38	93.47	93.64
MnasNet	93.78	95.26	94.51	94.80	95.15
CellViT	95.10	96.23	95.66	95.17	96.08
Proposed	97.81	98.08	97.94	97.81	97.50

Table 4 shows that on the Kvasir dataset, InceptionV3 and MobileNetV3 achieved an F1-score of 88.42% and 84.74%, respectively. At the same time, ResNet50 and YOLOV9 precision values are 90.25% and 93.78%, respectively. Furthermore, MnasNet and CellViT recall values are 95.26% and 96.23%, respectively. The CDCTNet obtained the highest recall and F1-score of 98.08% and 97.94%, respectively.

### 5.2. The loss

**Fig 5:**
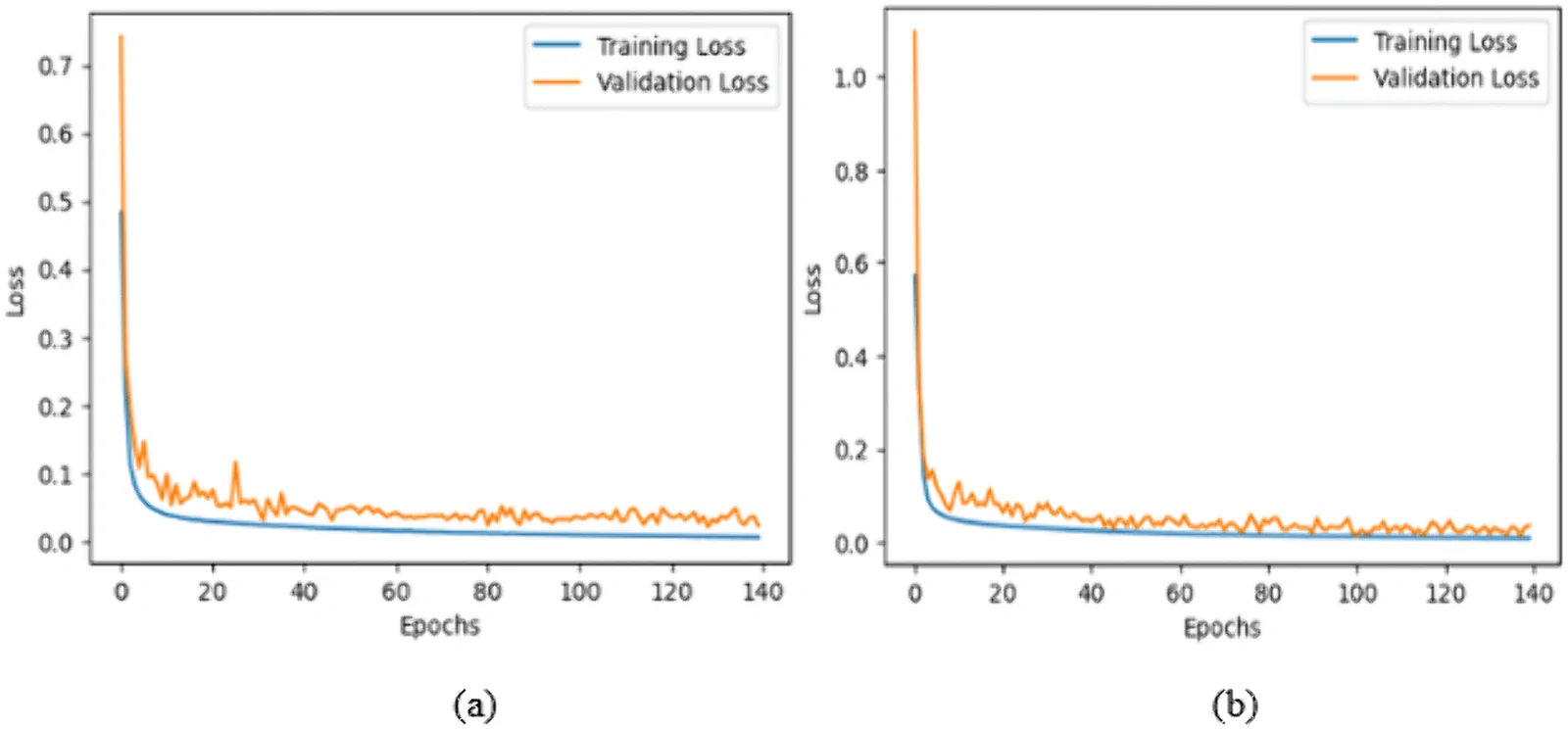
Training and validation loss (a) Kather and (b) Kvasir datasets for 140 epochs.

The training and validation loss is important in tracking the model performance at each epoch. We plotted the loss for each epoch depicted in Fig 5. Figure 5(a) shows that the initial validation loss on the Kather dataset is more than 0.7, and the training loss is below 0.5. After 60 epochs, it is close to 0.1. Moreover, on the Kvasir dataset, initial validation and training loss is high. Moreover, after 40 epochs, it started decreasing and reached close to zero.

### 5.3. Comparison with SOTA using ROC

**Fig 6:**
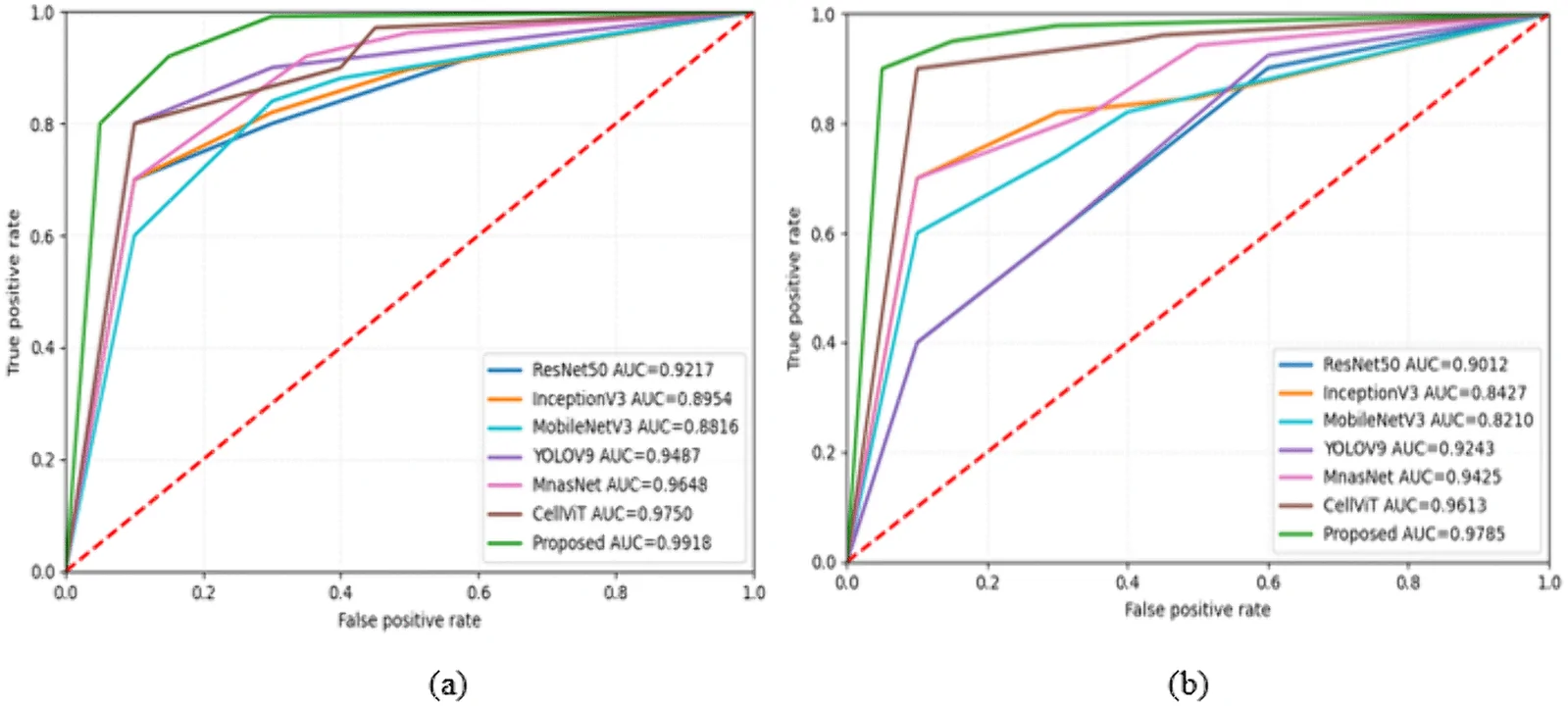
ROC Plot of based comparison on (a) Kather and (b) Kvasir datasets with SOTA methods.

We compare the proposed model AUC (area under the curve) value by true positive and false positive rates. Furthermore, we compared proposed model AUC value with ResNet50, Inception V3, MobileNetV3, YOLOV9, MnasNet and CellViT shown in Fig 6. Figure 6(a) shows that the AUC value of the InceptionV3 and MobileNet V3 is below 0.9. At that same time, ResNet50 and YOLOV3 have an AUC value of 0.9012 and 0.9243, respectively. Meanwhile CellViT and MnasNet achieved 0.9613 and 0.9425. Moreover, the proposed CDCTNet has an 0.9785 AUC value. Furthermore, the InceptionV3 and MobileNet V3 have an AUC value of 0.8954 and 0.8816 on Kvasir dataset. The ResNet50 and YOLOV3 obtained 0.9217 and 0.9487, respectively. At the same time, the CellViT has the second highest AUC value and CDCTNet obtained the highest AUC value of 0.9918.

### 5.4. Ablation study

In this section, we present an ablation study of the proposed model on the Kather and Kvasir datasets, and the result is presented in Table 5. Table 5 shows that with CNN, a precision value of 87.34% and Kappa score of 86.12% on the Kather dataset. At the same time, ViT achieved an improvement of 2.94% and 2.42% in the precision and F-score values. When we performed two-scale feature fusion using ViT and CNN, it obtained 92. 82% F1-score and 93.56% precision. With the inclusion of IEM for feature interaction between CNN and ViT, our model achieved the highest precision value of 95.02%. On the Kvasir dataset, CNN achieved 87.08% Kappa and 89.92% precision. At the same time, ViT obtained 90.15% Kappa and 92.04% precision. Meanwhile, CNN + ViT enhanced the precision and kappa scores by 3.14% and 4.15%, respectively. Moreover, CNN + ViT + IEM obtained the highest precision and F1-score of 97.81% and 97.94%, respectively, on the Kvasir dataset.

**Table 5:** Performance evaluation using different components.

Dataset	Components	Kappa	Precision	F1-score
**Kather**	CNN	86.12	87.34	86.89
	ViT	88.54	90.28	89.76
	CNN + ViT	92.19	93.56	92.82
	CNN + ViT + IEM	95.02	96.60	96.10
**Kvasir**	CNN	87.08	89.92	88.09
	ViT	90.15	92.04	91.68
	CNN + ViT	94.30	95.18	94.87
	CNN + ViT + IEM	97.50	97.81	97.94

### 5.5. Effect of patch size

**Fig 7:**
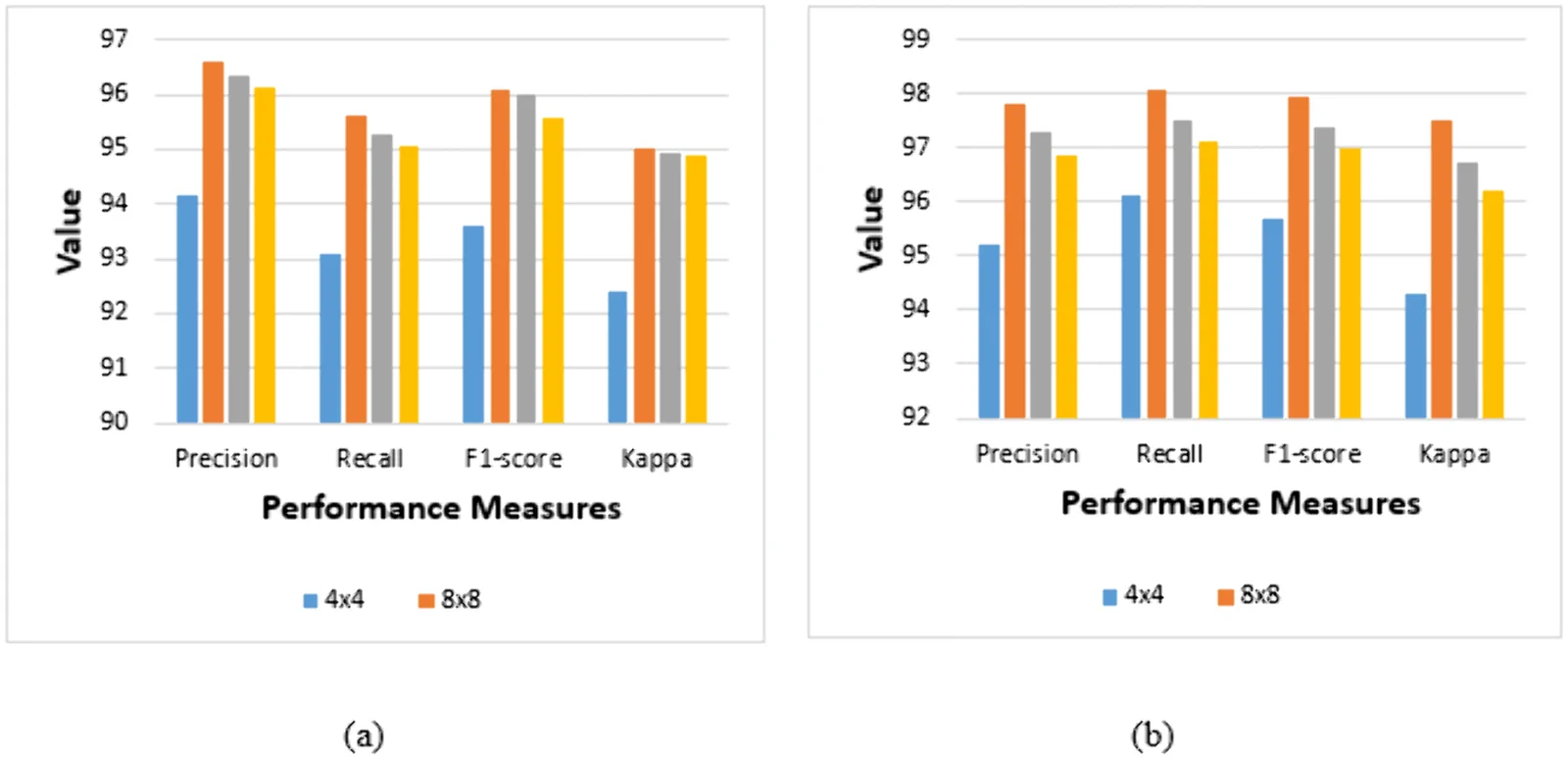
Effect of patch size on (a) Kather and (b) Kvasir datasets.

This section presents the effect of patch size on the performance. We experimented with patch sizes 4x4, 8x8, 16x16 and 32x32 in the ViT on the Kather and Kvasir datasets. The barplot-based performance analysis is depicted in Fig 7. Figure 7 (a) shows that a batch size 4x4 model has the lowest precision and F1-score of 94.17% and 93.62%, respectively. In addition, a smaller patch size model took a high computation time. We increased the patch size to 8x8, with this patch size model achieving the highest precision, a Kappa score of 96.60% and 95.02%, respectively. Furthermore, we tested the model with a patch size 16x16, and this parch size model had slightly less performance and computation costs. Moreover, with a patch size of 32x32, the model showed degraded performance measures. In Figure 7(b), we notice that with a batch size 4x4 model, the lowest recall and F1-score were 96.14% and 95.67%, respectively. Our model achieved the highest performance indicators. With the increase in patch size, model performance is less compared to 8x8 patch size.

### 5.6 Cross-sensor based performance analysis

**Fig 8:**
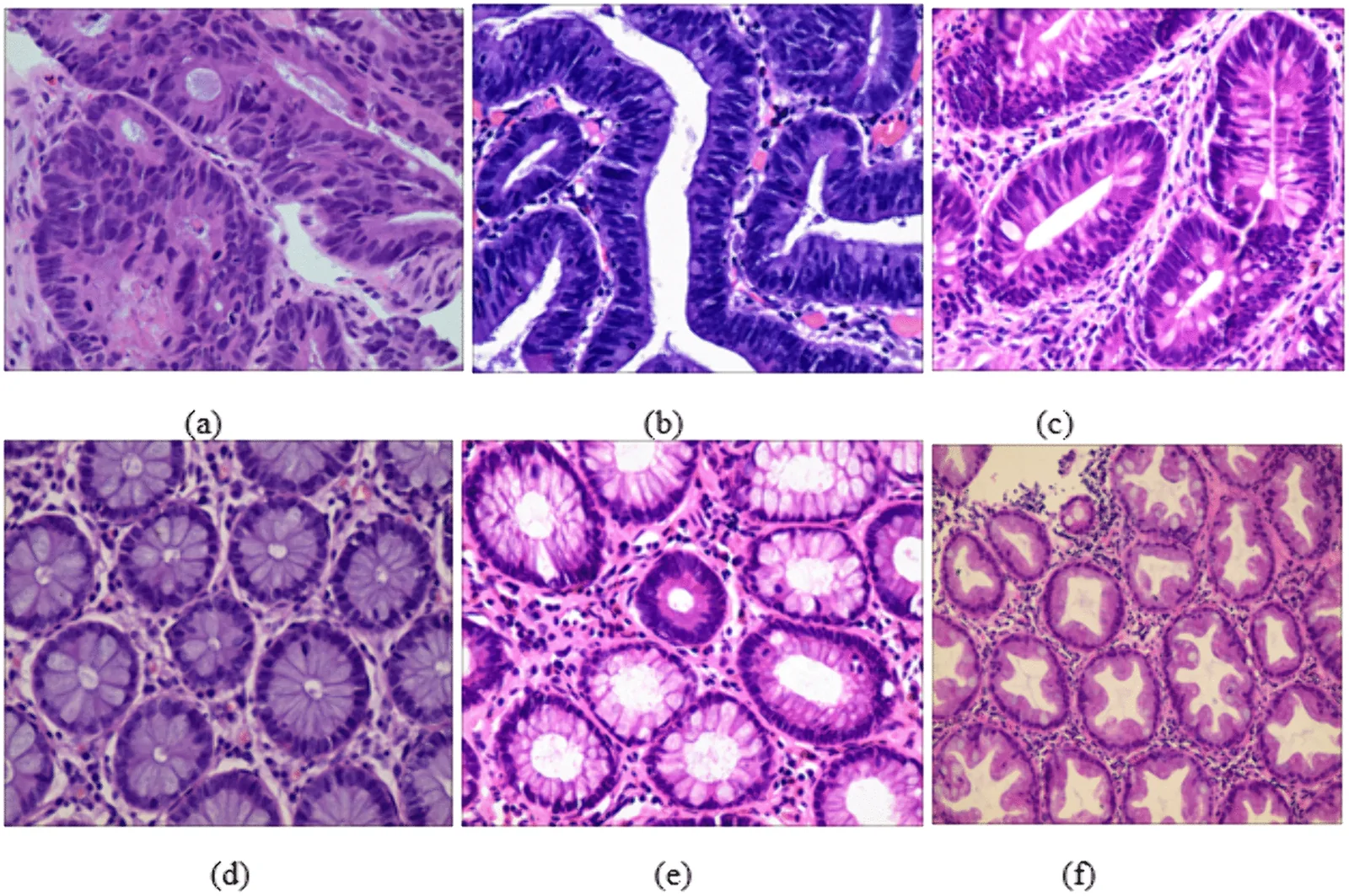
Sample images of EBHI dataset (a) Adenocarcinoma (b) High-grade IN (c) Low-grade IN (d) Normal (e) Polyp and (f) Serrated adenoma.

We evaluated model performance using cross sensor dataset, and results are presented in Table 6. In this experiment model is trained on the Kather dataset and tested on the EBHI (Histopathological Hematoxylin and Eosin Image) dataset (https://figshare.com/articles/dataset/EBHI-SEG/21540159/1) [37]. The EBHI dataset contains histopathological images of six classes, including Adenocarcinoma, High-grade in, Low-grade IN, Normal, Polyp and Serrated adenoma, with 795, 186, 639, 76, 474 and 58 images. Sample images for each class are shown in Fig 8.

**Table 6:** Results of the model on the EBHI dataset.

Dataset	Precision	Recall	F1-score	Accuracy	Kappa
EBHI	93.58	94.86	94.21	94.98	93.75

Furthermore, the experimental condition is kept the same as discussed in section 4.2 to test the model performance on the 20% dataset from each class of the EBHI dataset. We can notice in Table 6 that our model achieved 93.58% precision and 94.21% F1-score. In addition, the model obtained accuracy and Kappa scores of 94.98% and 93.75%, respectively.

**Fig 9:**
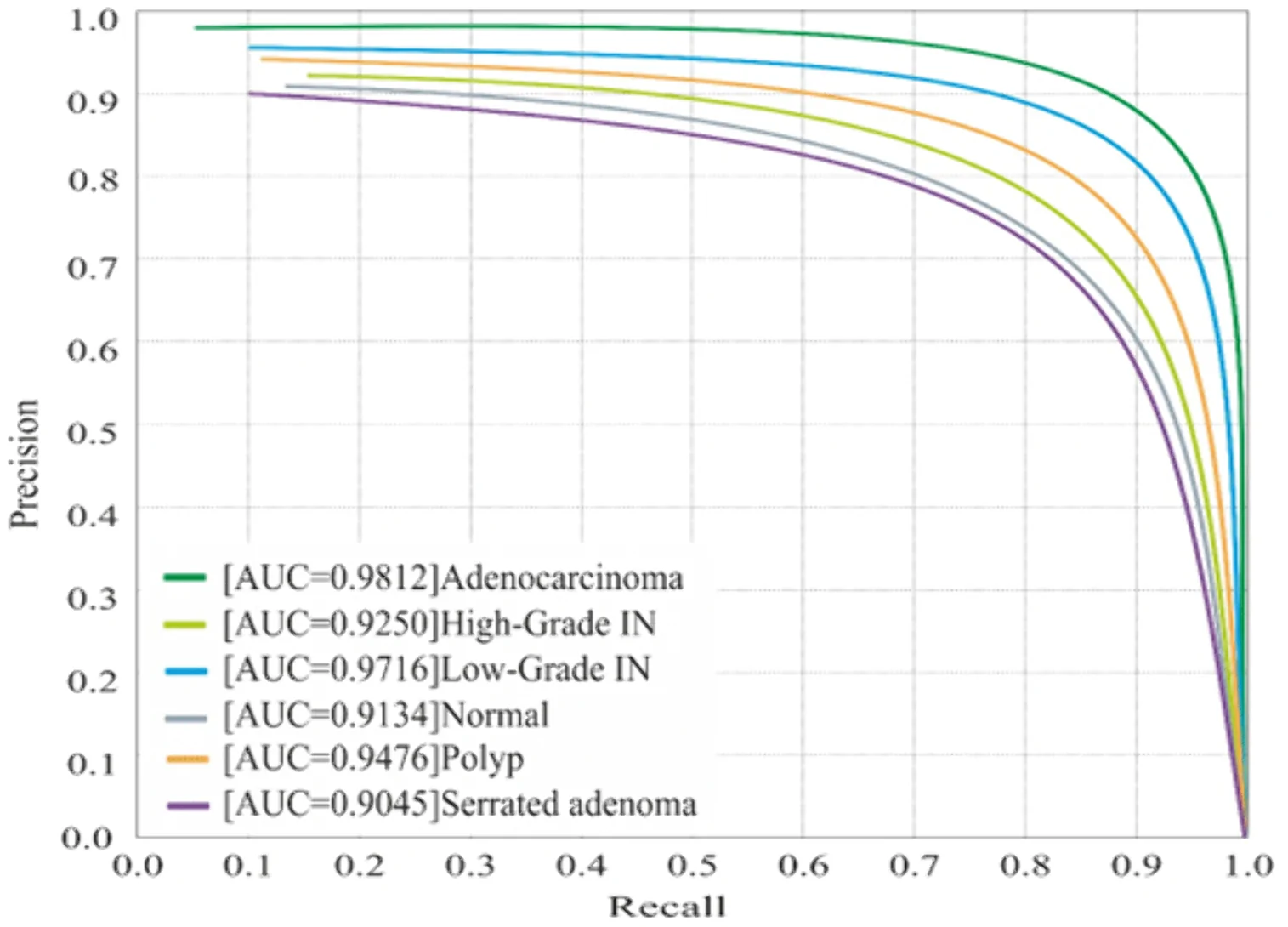
PR-curve based AUC analysis on EBHI dataset of the proposed method.

The sample in each class of the EBHI dataset is highly unbalanced. We plotted the PR (precision recall)-curve to analyze the model's performance in each class. Figure 9 shows that the Adenocarcinoma class obtained the highest AUC (area under the curve) value of 0.9812. At the same time, the High-grade IN class has 0.9250. Furthermore, the Low-grade in class obtained an AUC value of 0.9716. The fewer sample classes, such as Normal and Serrated adenoma, achieved an AUC value below 0.95. Moreover, the Polyp class has an AUC value of 0.9576.

### 5.7. The Grad-CAM based analysis

**Fig 10:**
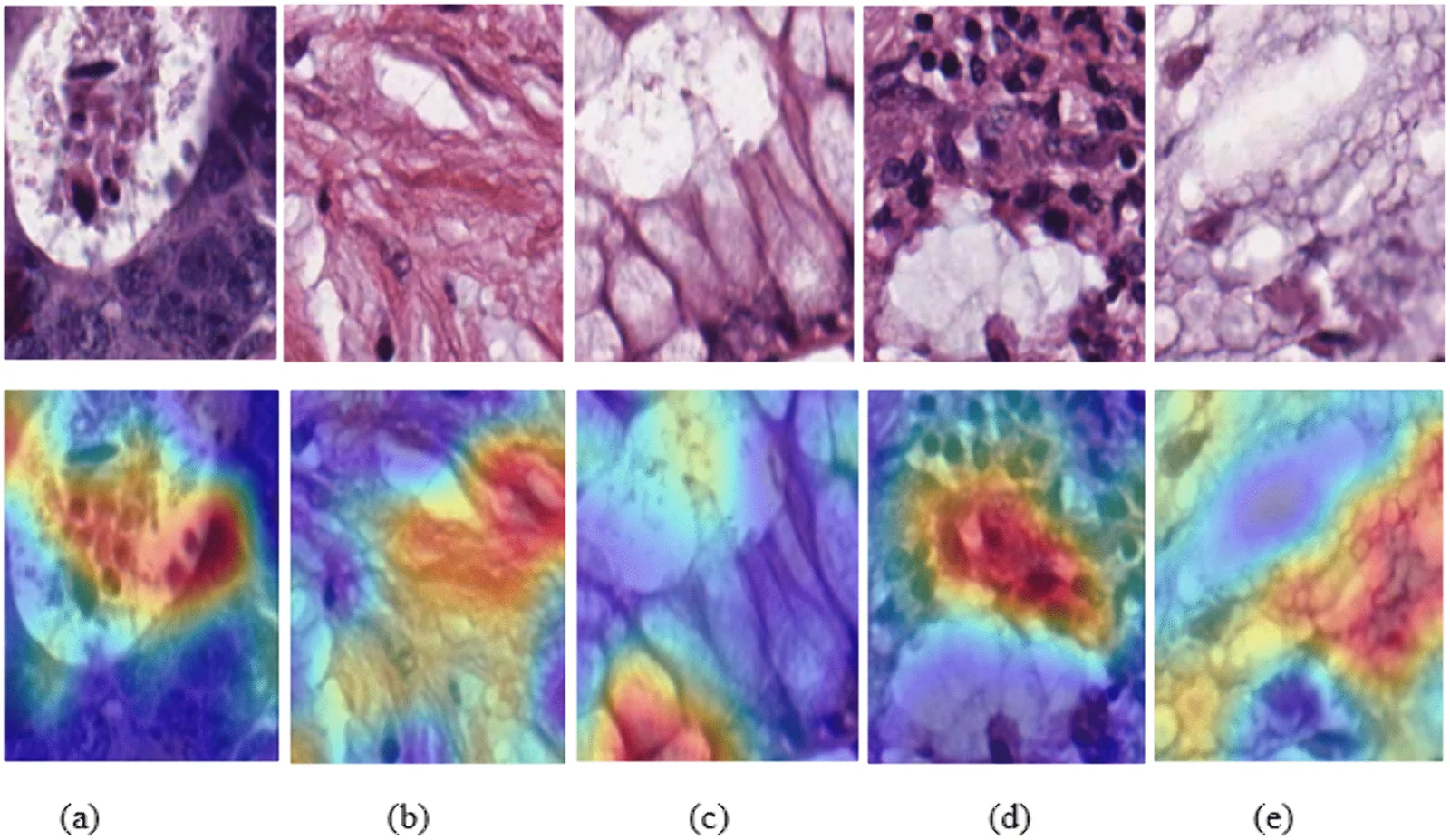
Sample image of the Kather dataset for Grad-Cam visualization (a) Tumor (b) Stroma (c) Mucosa (d) Lympho and (e) Debris.

The model decision process analysis is important for accurate disease diagnosis. In addition, the expert can use the decision process results to focus on the region of interest. We can see in Fig 10 that the Grad-CAM results of the model provide a deep hot spot area where the probability of the disease is high. Moreover, the light blue region indicates the possible region of the disease.

For the clinical workflow deployment, we will save the weight of the proposed model after training in the  .h5 file format (for example “Test.h5”). After that, this weight can be loaded to any web based application or cloud environment and new image of the colorectal cancer can be tested. Furthermore, the decision process explanation of results will be provided by the Grad-CAM module to the expert.

### 5.8. Ablation study using different hyper parameters

We performed an ablation study using different value of the loss hyper parameters and results of the kather dataset is presented in Table 7.

**Table 7:** Performance evaluation using different loss parameters on the kather dataset.

η	α	β	Precision	Accuracy	Kappa
0.05	0.5	0.5	96.01	95.13	94.93
0.10	0.5	0.5	96.60	95.60	95.02
0.15	0.5	0.5	96.15	95.27	94.96
0.20	0.7	0.3	95.98	94.87	94.16

In Table 7, we can see that with the isotropic loss weight 0.05, cosine similarity and Euclidian distance term each of 0.5, CDCTNet obtained precision of 96.01%. At the same time, isotropic loss weight value of 0.10 showed significant improvement in the precision value. When we further increase the value slight decrease in precision and kappa score is obtained. Moreover, the combination of η =0.20, α=0.7 and β=0.3 resulted 95.98% precision and 94.16% kappa score. These findings suggest that assigning excessive weight to the isotropic loss or placing greater emphasis on the cosine similarity component does not further improve feature discrimination.

## 6. Conclusion

In this work, we proposed CDCTNet, a hierarchical model for colorectal disease detection. Our model utilized two convolution blocks for the lesion's local high-dimensional spatial features. In addition, the ViT encoder provides the global correlation of the spatial feature map. Furthermore, we designed an IEM block to interact with the features between the convolution block and the ViT encoder. The CDCTNet results on Kather and Kvasir datasets are compared with SOTA methods. Our model achieved a precision and Kappa score of 96.60% and 95.02%, respectively. At the same time, CDCTNet obtained recall and F1 scores of 98.08% and 97.94%, respectively. The computation cots of the attention mechanism can be reduced and it should be tested on diverse real-time colorectal disease dataset for further validation. In addition, the patient identifiers are not available in these datasets, due to this patient level train and test has not be performed for experiment.The future study will explore the lightweight attention mechanism and feature optimization algorithm for the improvement of the classification.
